# Urinary small extracellular vesicles derived CCL21 mRNA as biomarker linked with pathogenesis for diabetic nephropathy

**DOI:** 10.1186/s12967-021-03030-x

**Published:** 2021-08-17

**Authors:** Ye Feng, Xin Zhong, Hai-Feng Ni, Cui Wang, Tao-Tao Tang, Li-Ting Wang, Kai-Yun Song, Ri-Ning Tang, Hong Liu, Bi-Cheng Liu, Lin-Li Lv

**Affiliations:** Institute of Nephrology, Zhongda Hospital, Southeast University School of Medicine, 87 Ding Jia Qiao Road, Nanjing, 210009 Jiangsu China

**Keywords:** Extracellular vesicles, Exosomes, Diabetic nephropathy, Biomarker, CCL21, Urine

## Abstract

**Background:**

Diabetic nephropathy (DN) is a leading cause of renal failure, whereas the effective and early diagnostic biomarkers are still lacking.

**Methods:**

Fourteen cytokines and chemokines mRNA were detected in urinary extracellular vesicles (EVs) from the screening cohort including 4 healthy controls (HC), 4 diabetes mellitus (DM) and 4 biopsy-proven DN patients, and was validated in another 16 HC and 15 DM and 28 DN patients. Correlation analysis was performed between the candidate biomarkers and clinic parameters as well as kidney histological changes. The findings were also confirmed in DN rat model with single injection of STZ.

**Results:**

The number of small EVs secreted in urine was increased in DN patients compared to DM patients and healthy controls, with expression of AQP1 (a marker of proximal tubules) and AQP2 (a marker of distal/collecting tubules). Small EVs derived CCL21 mRNA increased significantly in DN patients and correlated with level of proteinuria and eGFR. Interestingly, elevated CCL21 mRNA from urine small EVs was observed in DN patients with normal renal function and could discriminate early DN patients from DM more efficiently compared to eGFR and proteinuria. CCL21 also showed an accurate diagnostic ability in distinguishing incipient from overt DN. Histologically, CCL21 mRNA expression increased progressively with the deterioration of tubulointerstitial inflammation and showed the highest level in nodular sclerosis group (class III) in DN patients. Remarkable infiltration of CD3 positive T cells including both CD4 and CD8 positive T cell population were observed in DN patients with high-CCL21 expression. Besides, accumulation of CD3 positive T cells correlated with level of urinary small EVs derived CCL21 and co-localized with CCL21 in the tubulointerstitium in DN patients. Finally, the correlation of CCL21 expression in renal cortex and urinary small EVs was confirmed in STZ-induced DN rat model.

**Conclusions:**

Urinary small EVs derived CCL21 mRNA may serve as early biomarker for identifying DN linked with pathogenesis. CCL21 mRNA mediated T cell infiltration may constitute the key mechanism of chronic inflammation in DN.

## Background

Diabetic nephropathy (DN) develops in approximately 40% of patients with diabetes mellitus (DM), which is the leading cause of chronic kidney disease (CKD) and end-stage kidney disease worldwide [1]. Although estimated glomerular filtration rate (eGFR) and albuminuria are well established biomarkers for DN, they are not sufficient in patients with the absence of sustained albuminuria or preserved eGFR [2]. Hence, there is a pressing need for accurate and reliable markers to identify early renal dysfunction and structural lesions for DN [3, 4]. Furthermore, new biomarkers may also provide a better insight into the complicated pathophysiological processes for DN [5].

Regarding to biomarker development, detecting the molecules that are causal in DN, or markers of glomerular and tubular features and dysfunction might represent a rational approach [4]. Previously, metabolic and hemodynamic factors were thought to be the primary instigators of DN. However, accumulating evidence points to the critical roles of the immune response and inflammation lately [6, 7]. Recently, association between these inflammatory biomarkers and renal outcomes in DN has been reported [8]. Proteomic studies have revealed molecular signatures of inflammatory mediators in urine from patients with early, uncomplicated diabetes [9], which suggested that early appearance of inflammatory markers in urine may provide important clues for early diagnosis of DN. Chemokines and cytokines are key players in various inflammatory processes, ranging from immune cell development, initiation of immune responses and recruitment of immune cells. Several members of the inflammatory chemokine family were found to be involved in DN pathogenesis, particularly CCL2, CX3CL1, CCL5 [6]. Promising candidate biomarkers related to inflammation include circulating TNF-a and TNFR1 and TNFR2 and urine MCP/CCL [7]. A recent study also found that urinary CXCL16 and endostatin could reflect the degree of interstitial fibrosis and tubular atrophy, which were served as independent risk factors for progression in patients with advanced DN [10]. However, the potential of urinary chemokines and cytokines as biomarkers of DN and the implication in the pathogenesis of DN remained to be explored.

Extracellular vesicles (EVs), such as exosomes and microvesicles, are small membrane structures secreted by prokaryotic and eukaryotic cells. They are present in biological fluids and participate in multiple physiological and pathological processes [11]. Urinary EVs incorporate various cell-specific components (proteins, mRNAs) from all regions of the nephron, especially cells from endothelium cells, podocytes, epithelial cells facing urinary tract [12]. Thus, urinary EVs have gained significant interest as potential diagnostic biomarkers in renal diseases [13]. In recent years, increasing studies suggested that RNA was one of the important functional cargo in EVs since the first report in 2007, which received substantial attention as a novel mechanism of genetic exchange between cells [14–16]. Moreover, according to its stability with the protection of vesicles, it has been soon recognized that EV RNAs might also have utility as disease biomarkers [17]. Our previous studies have shown that urinary exosomal CCL2 mRNA, miRNAs could reflect the deterioration of kidney function as well as histological change for CKD patients [18, 19]. Studies of urinary EVs derived RNAs as biomarker of DN, especially its role in assessing structural damage of kidney and its link with pathogenesis are still needed.

In this study, we aimed to identify the potential candidate biomarkers of DN through the detection of inflammatory cytokines and chemokines in urinary small EVs, and further explore the relationship of the candidate biomarkers with histological changes in DN patients. Due to the mixed population of the vesicles, we use the term small EVs to define the fraction isolated through ultracentrifugation at 2,00,000*g* [20]. Patients with biopsy-proven DN and T2DM were enrolled in the screening and validation cohorts. In addition, to validate the role of small EV-derived mRNA in DN progression, we established DN rat model with STZ injection. We revealed that urinary CCL21 mRNA derived from small EVs was a promising marker of renal dysfunction and histological damage for DN.

## Methods

### Patients and controls

The study was approved by the Ethics Committee of Zhongda Hospital, Southeast University. Two cohorts of patients were enrolled in the study. A group of 4 healthy controls, 4 patients with diabetes mellitus (DM) and 4 patients with biopsy-proven diabetic nephropathy (DN) were included in the screening cohort. Normal kidney tissues obtained from patients with renal carcinoma were used as controls. In the validation cohort, 16 healthy controls and 15 patients with DM and 28 patients with DN proven by renal biopsy were enrolled. The exclusion criteria of both cohorts were shown as following: subjects with severe liver injury or malignancy, renal replacement therapy, including renal transplantation or dialysis, acute or chronic infection and pregnant women. Informed consent was obtained from all participants and first morning urine was collected. All the laboratory measurements, including total cholesterol, triglyceride, blood urea nitrogen (BUN), serum creatinine (SCr), uric acid (UA), plasma albumin, urinary ACR and urinary creatinine, were obtained on the day of urine sample collection. Estimated glomerular filtration ratio (eGFR) were calculated by the Chronic Kidney Disease Epidemiology Collaboration (CKD-EPI) formula.

### Animal studies

All animal studies were performed in accordance with the protocols approved by the Ethics Committee of Southeast University. Sixteen 8–10-week old male Sprague Dawley (SD) rats weighting 250–300 g purchased from National Model Animal Center of Nanjing University were used. All the rats were fed on standard rat chow and provided with water ad libitum. Rats received a single intraperitoneal injection of streptozotocin (STZ, 55 mg/kg of body weight, Sigma-Aldrich, USA) or vehicle (sodium citrate buffer, pH 4.5) randomly after 12 h-fasting. One week after injection, STZ-injected rats with a plasma glucose concentration above 16.67 mmol/L were selected for the study (n  =  8). Rats with vehicle injection were enrolled as control group (n  =  8). Due to overt behavioral activity, the experiments could not be blinded to whether the rats were injected with STZ or vehicle. All rats were sacrificed and renal tissues were collected at week 20 post-injection. 24-h urine samples were collected with a rat metabolic cage. Albuminuria was measured using a rat albumin-specific ELISA kit (Jiancheng bioengineering, China) and the creatinine concentration and blood urea nitrogen (BUN) level were assessed with the assay kit (Jiancheng Bioengineering, China).

### Small EVs isolation and characterization

The first morning urine samples were collected from patients and healthy controls. For animal study, 24-h urine samples were collected from rats with or without STZ-treatment. Small EVs were isolated by differential centrifugation as previously described [18]. Briefly, 25 ml urine samples were centrifuged at 3000*g* and 13,500*g* for 20 min at 4 °C to remove cellular debris and large extracellular vesicles, respectively, and followed by a further ultracentrifugation at 2,00,000*g* for 2 h at 4 °C. The pellets were suspended by PBS and stored at −80 °C for further experiments.

For small EVs characterization, EVs pellet was detected by electron microscopy. Briefly, the pellets were applied to nickel grids and stained with 2% phosphotungstic acid for 5 min and detected through Hitachi HT 7700 (Japan) transmission electron microscope at 80 kV. Nanoparticle tracking analysis (NTA) was performed with the ZetaView PMX 110 (Particle Metrix, Meerbusch, Germany). Briefly, purified small EVs were diluted with PBS buffer and the particle size and concentration were measured. The data was analyzed by corresponding software, ZetaView 8.04.02. Dilution factors and resuspension volumes were used to convert the yield from concentration to an accurate number of particles, and the quantification was normalized to urine creatinine.

For western blot analysis, the pellets from equal volume of urine were lysed and loaded on 10% polyacrylamide gels and transferred to the membrane. Antibodies against exosome markers, including mouse anti-Alix (Santa Cruz, sc-53540), rabbit anti-CD81 (Cell Signaling Technology, 56039 for human samples, abcam, ab109201 for rat samples), anti-CD63 (Abcam, ab213090 for human samples, santa cruz, sc-15363 for rat samples) and tubule marker anti-AQP1(Aquaporin1) (Santa Cruz, sc-32737) and anti-AQP2(Aquaporin 2) (Santa Cruz, sc515770) were diluted (1:1000) to incubate the membrane overnight. Secondary antibodies and enhanced chemiluminescence detection reagents were applied for detection. The quantification was normalized to urine creatinine.

To validate the presence of CCL21 mRNA inside small EVs, RNase A digestion with or without Triton X-100 was performed. Purified urinary small EVs were incubated with 2 mg/ml of RNase A (Beyotime) at 37 °C for 30 min, followed by treatment of RNase inhibitor (10  ×  concentrated, Beyotime, China). When both RNase A and Triton X-100 treatments were applied, purified urinary small EVs were incubated with 1% Triton X-100 (Beyotime) for 30 min at 37 °C firstly, after which RNase A was added.

### mRNA isolation and RT-PCR

Total RNA isolation was performed using TRIzol following the manufacture’s protocol (TAKARA, Japan). mRNA was reverse transcribed using PrimeScript RT reagent Kit (TAKARA, Japan) and PCR was conducted with Sybr Premix Ex Taq performing in 7300 Real-Time PCR System (Applied Biosystems, US). The data was normalized to the expression of GAPDH. Primer sequences are shown in Table 1.

**Table 1:** Primers for quantitative RT-PCR

Primer	Forward (5′–3′)	Reverse (5′–3′)
Homo CCL-2	CCTTCATTCCCCAAGGGCTC	GGTTTGCTTGTCCAGGTGGT
Homo CCL-5	CGCTGTCATCCTCATTGCTA	CCAGACTTGCTGTCCCTCTC
Homo CCL-21	AGAGAGACCGAGGAGGGAGA	TTTGGGTGGGAAGACAGAAC
Homo CCL-22	CTGTTCCCATCAGCGATTCC	GACCCAGAAGTGGGATGTGT
Homo CXCL-1	ACTCAAGAATGGGCGGAAAG	TCCTAAGCGATGCTCAAACA
Homo CXCL-2	CTCAAGAATGGGCAGAAAGC	AAACACATTAGGCGCAATCC
Homo CCR2	CGGTGCTCCCTGTCATAAAT	CCCAAAGACCCACTCATTTG
Homo IL-10	CCACGCTTTCTAGCTGTTGA	CTCCGAGACACTGGAAGGTG
Homo IL-18	CAGACCTTCCAGATCGCTTC	ATAGAGGCCGATTTCCTTGG
Homo IL-22	AGGCTCAGCAACAGGCTAAG	TTCAGCTTTGCTCTGGTCAA
Homo IFN-γ	AACTAGGCAGCCAACCTAAGC	AAGCACCAGGCATGAAATCT
Homo IL-1β	GCATCCAGCTACGAATCTCC	TCGTTATCCCATGTGTCGAA
Homo IL-6	CCTTCCAAAGATGGCTGAAA	AGCTCTGGCTTGTTCCTCAC
Homo TNFα	CCCAGGGACCTCTCTCTAATC	TGAGGTACAGGCCCTCTGAT
Homo GAPDH	CTCTGCTCCTCCTGTTCGAC	GCGCCCAATACGACCAAATC

### Immunohistochemistry and immunofluorescence staining

Paraffin-embedded kidney tissue sections were used for immunohistochemistry. Briefly, the sections were incubated with primary antibodies to CD3 (1:100, ab5690, Abcam, USA), CD4 (ZM-0418, ZSGB-Bio, China) and CD8 (ZA-0508, ZSGB-Bio, China) overnight at 4 °C and then analyzed with streptavidin peroxidase detection system (Maixin Technology Co., Ltd., China) according to the manufacturer’s protocol.

Immunofluorescence staining was performed to localize the expression of CCL21 and CD3 with tissue sections using anti-CCL21 (1:100, AF366, R&D Systems, USA) and anti-CD3 (1:100, ab5690, Abcam, USA) antibodies in a chamber overnight at 4 °C, followed by incubation with fluorescein-labeled secondary antibodies (Invitrogen, USA) for 1 h. DAPI was used for cell nuclei staining. Immuno-stained samples were visualized using a confocal microscope.

### Statistical analysis

Data are expressed as mean  ±  SD or median (IQR). A two-tailed unpaired Student’s t test or Mann–Whitney U test was used for comparison between two groups, and one-way ANOVA were performed for comparisons of data with more than two groups followed by Newman-Keils post-test. All analyses were carried out by GraphPad Prism 5.0. p  <  0.05 was considered statistically significant.

## Results

### Clinical characteristics of cases and controls

The demographics and clinical characteristics of the enrolled individuals are summarized in Tables 2, 3 for screening and validation cohort, respectively. Healthy controls, patients with T2DM and biopsy-proven DN were included in both cohorts. No significant differences were observed between groups including age, total cholesterol, triglyceride and uric acid (UA). Patients with T2DM and DN showed higher level of BMI than healthy controls. Compared to both healthy controls and patients with T2DM, DN patients showed lower level of eGFR, higher levels of blood urea nitrogen (BUN), serum creatinine (SCr) and Albumin Creatinine Ratio (ACR). In addition, plasma albumin concentration was reduced in patients with DN.

**Table 2:** Clinic characteristics of screening cohort

	Healthy control (n = 4)	T2DM (n = 4)	DN (n = 4)
Males	2 (50%)	2 (50%)	2 (50%)
Age (years)	31 (25–38)	55 (49–59)	51 (47–53)
BMI (kg/m^2^)	23.67 (21.68–26.31)	25.50 (20.57–29.73)	25.96 (20.02–32.12)
Total cholesterol (g/L)	–	5.08 (2.93–6.96)	4.66 (4.22–5.15)
Triglyceride (mg/dL)	–	2.14 (1.17–3.34)	2.00 (1.68–2.20)
BUN (mmol/L)	4.9 (4.1–5.5)	7.1 (5.6–8.3)	12.4 (7.5–16.4)**#
SCr (umol/L)	70.5 (61.3–79.3)	81.3 (69.8–91.5)	161.5 (105.5–219.5)*#
UA (umol/L)	310 (294–324)	308 (269–365)	333 (308–375)
Plasma albumin (g/L)	48 (45–51)	42 (38–45)	31 (25–36)**#
eGFR (ml/min/1.73m^2^)	94 (81.3–112.5)	90 (81.3–101.3)	57.8 (48.9–67.4)**#
Urinary ACR (mg/g)	2.4 (0–8.9)	55 (5–150)	579 (208–707)**##

eGFR has been calculated using the CKD-EPI Creatinine formula (ml/s per 1.73 m2). Values are expressed as median (IQR) (Mann–Whitney U test, *p  <  0.05, **p  <  0.01 compared with healthy controls; #p  <  0.05, ##p  <  0.01 compared with T2DM)

*BMI* body mass index; *BUN* blood urea nitrogen; *SCr* serum creatinine; *UA* uric acid; *eGFR* estimated glomerular filtration rate

**Table 3:** Clinic characteristics of validation cohort

	Healthy control (n = 16)	T2DM (n = 15)	DN (n = 28)
Males	8 (50%)	8 (53%)	14 (50%)
Age (years)	38 (25–58)	51.8 (40.5–67.6)	59 (51–69)
BMI (kg/m^2^)	22.1 (20.62–23.71)	25.69 (23.60–28.52) *	24.48 (22.12–27.99)*
Total cholesterol (g/L)	–	4.71 (4.19–5.67)	4.48 (3.68–6.89)
Triglyceride (mg/dL)	–	2.55 (1.59–3.97)	1.89 (1.47–2.32)
BUN (mmol/L)	4.15 (3.53–4.96)	5.99 (4.97–9.1)	12.3 (6.9–17.7)***##
SCr (μmol/L)	78 (68.25–89.5)	79 (68.4–111)	188 (99–254)**#
UA (μmol/L)	307 (274–341)	329 (294–387)	372 (318–495)
Plasma albumin (g/L)	45 (40–50)	42 (40–43)	30 (26.5–35.5)****####
eGFR (ml/min/1.73m^2^)	86.2 (78.7–97.3)	83.8 (60.2–99.5)	31.8 (18.7–73.6)*#
Urinary ACR (mg/g)	4 (0–28)	43 (2–159)	698 (399–1056)**##

Values are expressed as median (IQR) (Mann–Whitney U test, *p  <  0.05, **p  <  0.01, ***p  <  0.001, ****p  <  0.0001 compared with healthy controls; #p  <  0.05, ##p  <  0.01, ####p  <  0.0001 compared with T2DM)

### Screening and validation of cytokines and chemokines expression in urinary small EVs

**Fig. 1:**
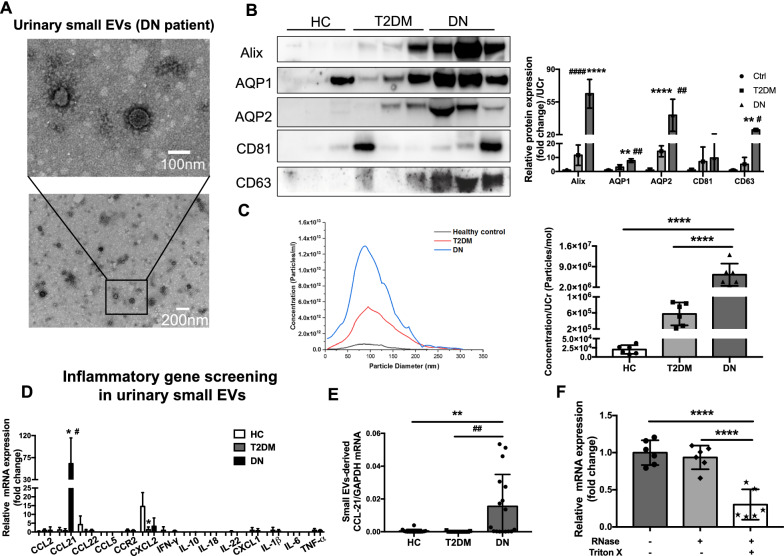
Urinary small EVs derived CCL21 mRNA increases in biopsy-proven DN patients. **A** Representative micrographs of transmission electron microscopy showed the typical size and shape of urinary small EVs from DN patients with higher and lower magnification. (scale bar: up-100 nm, down-200 nm). **B** Western blot and analysis of exosome (Alix, CD81 and CD63) and tubular marker AQP1 and AQP2 in urinary small EVs fraction from healthy individuals, patients with T2DM and biopsy-proven DN and the quantification was normalized to urine creatinine. **C** Size distribution and particle number of urinary small EVs analyzed by nanoparticle tracking analysis (NTA) and the quantification was normalized to urine creatinine. **D** Fourteen cytokines and chemokines as well as receptors were screened in DN (n  =  4 for each group). The relative expression of CCL21 mRNA in urinary small EVs showed significant increase in DN patients compared with healthy controls and T2DM patients. **E** The expression of CCL21 mRNA in urinary small EVs of DN patients (n  =  28) was significantly higher than healthy controls (n  =  16) and patients with T2DM (n  =  15) in validation cohorts. *p  <  0.05, **p  <  0.01, ***p  <  0.001, ****p  <  0.0001, compared with healthy controls. #p  <  0.05, ##p  <  0.01, ###p  <  0.001, ####p  <  0.0001, compared with patients with T2DM. *HC* healthy control; *T2DM* type 2 diabetes; *DN* diabetic nephropathy. **F** CCL21 mRNA expression in urinary small EVs isolated from DN patients with or without RNase and Triton X treatment. ****p  <  0.0001, compared with urinary small EVs treated with RNase and TritonX

Typical membrane morphology of urinary small EVs was shown by transmission electron microscopy (Fig. 1A). Western blot analysis revealed that the protein expression of small EV markers (Alix, CD81, CD63) was significantly increased in DN patients (Fig. 1B), indicating that a greater number of small EVs were secreted in urine of DN patients compared with T2DM patients and healthy controls (HC), which was confirmed by particle quantification through nanoparticle tracking analysis (Fig. 1C). Interestingly, the protein level of tubular marker AQP1 and AQP2 was detectable in urinary small EVs of DN patients (Fig. 1B), which suggested that renal tubules are one of the sources of urine small EVs.

Next, 14 key inflammatory cytokines and chemokines as well as their receptors, CCL2, CCL21, CCL22, CCL5, CCR2, CXCL2, IFN-r, IL-1β, IL-6, IL-10, IL-18, IL-22, TNFα and CXCL1 were detected. Impressively, CCL21 mRNA expression was found to be stably expressed with remarkable increased levels in patients with DN, compared to both healthy controls and T2DM patients (Fig. 1D). For further validation, CCL21 mRNA expression was analyzed in the cohorts including 16 healthy controls, 15 patients with T2DM and 28 patients with DN. Consistently, small EVs derived CCL21 mRNA showed remarkably increase in DN patients as compared with healthy controls and T2DM patients (Fig. 1E). Besides, to confirm that CCL21 mRNA is confined inside the urinary small EVs, EVs pellet was treated with RNase in the absence or presence of Triton X. The results showed that CCL21 mRNA expression in urinary small EVs from DN patients was not diminished after RNase treatment, but was sharply decreased after membrane disruption when both Triton and RNase were applied (Fig. 1F). Thus, urinary small EVs were increasingly secreted with enhanced CCL21 mRNA packaged inside the vesicles in DN patients compared to T2DM patients and healthy controls.

### Diagnostic performance of urinary small EVs derived CCL21 for DN

**Fig. 2:**
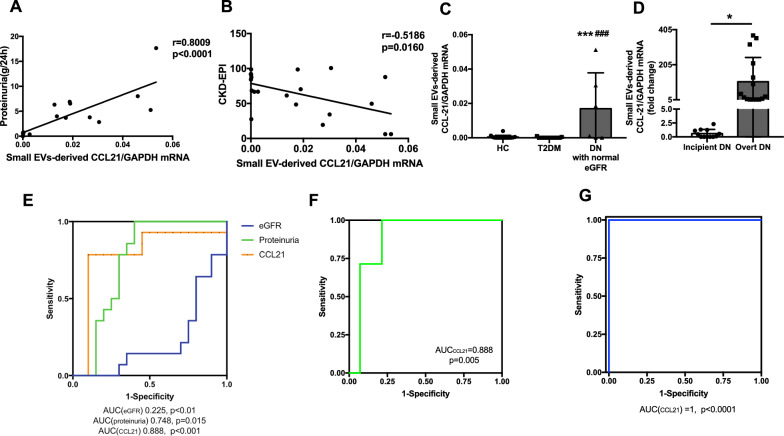
Diagnostic performance of urinary small EVs derived CCL21 mRNA for DN. **A** Urinary small EVs derived CCL21 mRNA was positively correlated to the levels of 24-h proteinuria at the time of renal biopsy (r  =  0.8009, p  <  0.0001). **B** Plots of correlation between urinary small EV derived CCL21 mRNA expression and eGFR calculated by CKD-EPI formula in DN patients (r  =  − 0.5186, p  =  0.0160). **C** The expression of urinary small EVs derived CCL21 mRNA of DN patients with normal eGFR (eGFR  >  90 ml/min/1.73m^2^) and T2DM patients, heathy controls. ***p  <  0.001 compared with healthy controls, ###p  <  0.001 compared with T2DM patients. **D** The expression of urinary small EVs derived CCL21 mRNA were remarkably increased in overt DN patients (ACR  >  300 mg/g creatinine, n  =  16) compared to incipient DN patients (ACR  <  300 mg/g creatinine, n  =  12) (*p  <  0.05). **E** ROC curve analysis of eGFR, proteinuria level and CCL21 level in urinary small EVs to discriminate DN patients from T2DM patients (AUC: eGFR, 0.225, 95% CI of 0.065–0.385, proteinuria, 0.748, 95% CI of 0.577–0.920, CCL21, 0.888, 95% CI of 0.752–1). **F** ROC curve analysis of CCL21 expression in urinary small EVs to discriminate DN patients with normal eGFR (eGFR  >  90 ml/min/1.73m^2^) from T2DM patients (AUC  =  0.888, 95% CI 0.737–0.997). **G** ROC curve analysis of CCL21 expression in urinary small EVs to discriminate incipient DN patient with overt DN patients (AUC: 1.0, 95% CI 1.0–1.0)

Correlation were evaluated between urinary small EVs derived CCL21 mRNA and clinical parameters in DN patients. A prominent positive correlation was found between urinary small EVs derived CCL21 mRNA and levels of 24-h proteinuria (r  =  0.8009, p  <  0.0001; Fig. 2A), and an inverse correlation with eGFR was found (r  =  − 0.5186, p  =  0.0160; Fig. 2B). Notably, DN patients with normal eGFR (eGFR  >  90 ml/min/1.73m^2^, n  =  6) showed remarkable elevation of urinary small EVs derived CCL21 mRNA compared with T2DM patients and healthy controls, indicating the earlier enhanced secretion of small EVs derived CCL21 mRNA than renal dysfunction (Fig. 2C). In addition, DN patients were defined as incipient DN with microalbuminuria (ACR 30–300 mg/g creatinine) (n  =  12) and overt DN with macroalbuminuria (ACR  >  300 mg/g creatinine) (n  =  16). Interestingly, small EVs derived CCL21 mRNA expression was much higher in overt DN group compared to incipient DN patients (Fig. 2D).

To evaluate the diagnostic ability of urinary small EVs derived CCL21 for DN, receiver operating characteristic (ROC) curves were generated. It was found that urinary small EVs derived CCL21 expression could discriminate patients with DN from patients with T2DM efficiently, with the area under the ROC curve (AUC) of 0.888 (95% CI 0.752–1) (Fig. 2E), which is more efficient than level of eGFR and proteinuria (AUC of eGFR, 0.225 with 95% CI 0.065–0.385, AUC of proteinuria, 0.748 with 95% CI 0.577–0.920). Interestingly, small EVs derived CCL21 expression could also distinguish DN patients with normal eGFR from T2DM patients with an AUC of 0.888 (95% CI 0.737–0.997) (Fig. 2F). Impressively, CCL21 showed very high diagnostic accuracy in discriminating incipient from overt DN (AUC  =  1, sensitivity  =  100%, specificity  =  100%; Fig. 2G). Thus, CCL21 mRNA derived from urinary small EVs may provide more efficient approach for identifying early DN from T2DM compared to eGFR and proteinuria.

### Urinary small EVs derived CCL21 mRNA in different stages of DN divided by histological damage

**Fig. 3:**
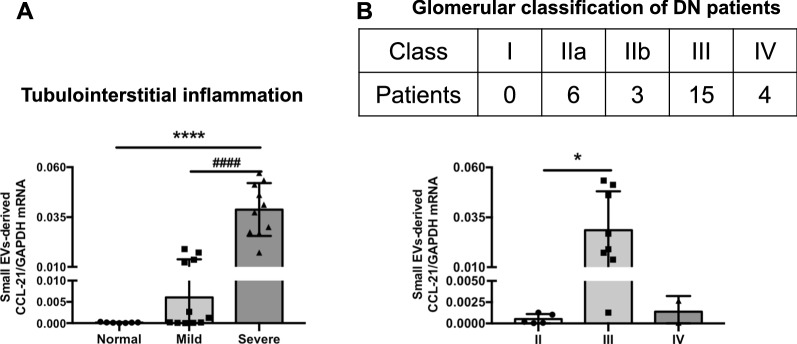
Urinary small EVs derived CCL21 mRNA in different stages of DN divided by histological damage. **A** The expression of CCL21 mRNA in urinary small EVs of DN patients with normal (0, n  =  7), mild (<  25%, n  =  11) or severe (>  25%, n  =  10) tubulointerstitial inflammation. Urinary small EVs derived CCL21 mRNA expression increased progressively with the deterioration of tubulointerstitial inflammation. (****p  <  0.0001, compared with normal group; ####p  <  0.0001, compared with mild group). **B** Urinary small EVs derived CCL21 expression in DN patients based on glomerular classification (*p  <  0.05 compared with patients with class II)

Next, the correlation between small EVs derived CCL21 mRNA with renal histological change was explored. Tubulointerstitial inflammation was semiquantitatively graded into normal (No significant infiltrations), mild (less than 25% of interstitium infiltration) and severe (> 25% of interstitium infiltration). We found that urinary small EVs derived CCL21 mRNA expression increased progressively with the deterioration of tubulointerstitial inflammation (Fig. 3A). The expression of CCL21 mRNA was analyzed in different stages of DN patients divided according to the glomerular lesions [21]. Interestingly, patients from class III, with nodular sclerosis, showed sharply increase in CCL21 mRNA expression compared with patients from class II (mild or severe mesangial expansion) and IV (advanced diabetic glomerulosclerosis) (Fig. 3B). Hence, enhanced level of urinary small EVs derived CCL21 mRNA was associated with histological damage of DN which may indicate a pathological role in the development of the disease.

### *CCL21 expression correlated with CD3* + *T cell infiltration in DN*

**Fig. 4:**
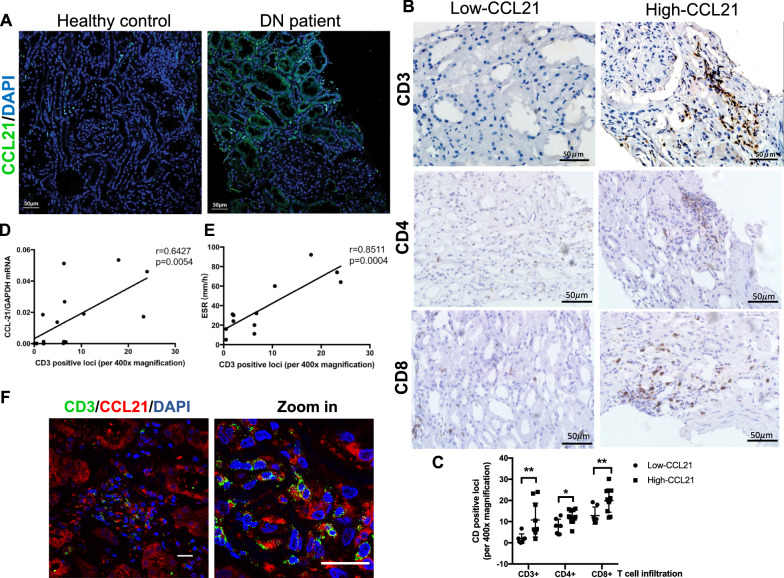
CCL21 expression correlated with CD3  +  T cell infiltration in DN. **A** Representative micrographs of immunofluorescent staining of CCL21 (green) in renal tissue from controls (normal kidney tissue from patients with renal carcinoma) and patients with DN. Nuclei were stained with DAPI (blue). Scale bar: 50 μm. Representative micrographs (**B**) and the quantification (**C**) of CD3-positive cells, CD4-positive cells and CD8-positive cells in renal tissues from DN patients with low (<  median) and high (>  or  =  median) expression of urinary small EVs derived CCL21. Scale bar: 50 μm. **D** The correlation between CD3-positive cells and the expression of urinary small EVs derived CCL21 (r  =  0.6427, p  =  0.0054). **E** The correlation between the number of CD3-positive cells with ESR levels in DN patients (r  =  0.8511, p = 0.004). **F** Representative micrographs of immunofluorescent staining for CD3 (green) and CCL21 (red) in renal tissue from patients with DN. Nuclei were stained with DAPI (blue). Scale bar: 20 μm

Next, we examined the expression of CCL21 in kidney biopsy in DN patients, normal kidney tissue from patients with renal carcinoma was used as the controls. It turned out that increased CCL21 expression was observed in both tubules and interstitial in DN patients compared to healthy controls (Fig. 4A). Since CCL21 is one of the most important chemokine signaling taking responsibility for homing of T cells, CD3 positive T cells was then detected in kidney tissue. DN patients were divided into Low-CCL21 and High-CCL21 group based on the median levels of urinary small EVs derived CCL21 mRNA. Notably, obvious accumulation of CD3 positive T cells, including CD4 and CD8 positive populations were observed in DN patients of High-CCL21 group (Fig. 4B, C). Interestingly, urinary small EVs derived CCL21 mRNA expression was positively correlated with CD3 positive loci in DN patients (r  =  0.6427, p  =  0.0054; Fig. 4D). In addition, CD3 positive loci in kidney was strongly correlated with erythrocyte sedimentation rate (ESR) levels, indicating the inflammatory status of DN patients (r  =  0.8511, p  =  0.0004; Fig. 4E). Impressively, co-localization of CD3 and CCL21 was observed in the tubulointerstitium in DN patients (Fig. 4F), suggesting the possible role of CCL21 in recruiting CD3 + T cell in DN. Thus, upregulation of urine small EVs derived CCL21 mRNA may reflect the increase of CCL21 expression in kidney, which correlated with the CD3 + T cell infiltration.

### Urinary small EVs derived CCL21 mRNA increases in STZ-induced DN rats and correlated with the alteration in kidney

**Fig. 5:**
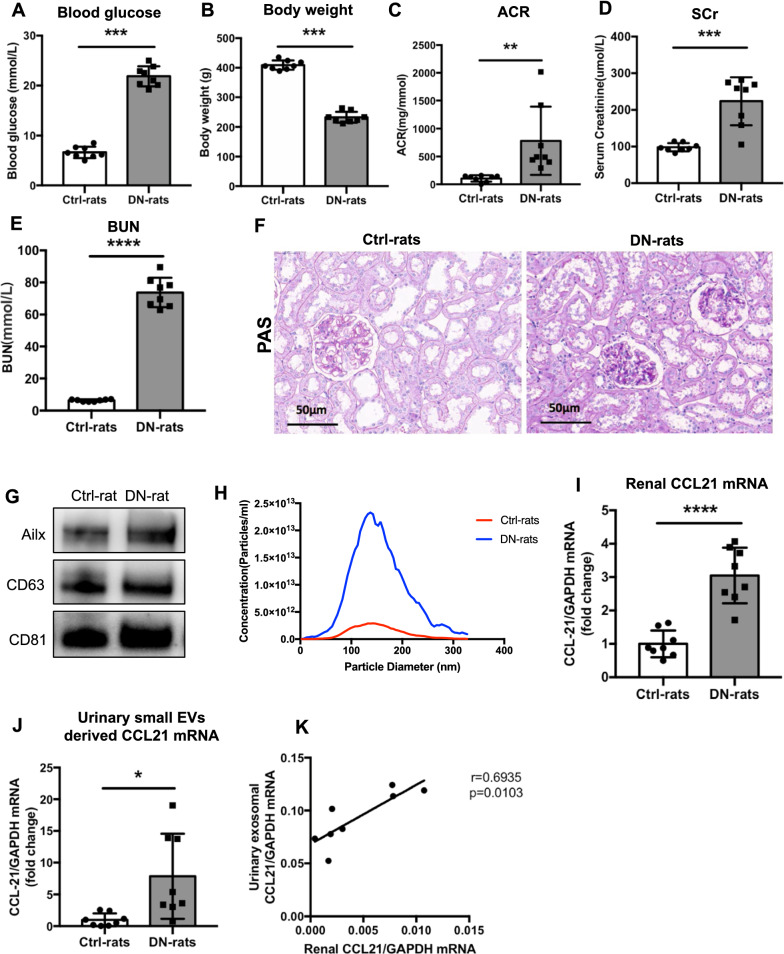
Urinary small EVs derived CCL21 mRNA increased in STZ-induced DN rats and correlated with the alteration in kidney. **A**, **B** Blood glucose and body weight of rats with or without STZ treatment at week 20 after injection. Significant increase of blood glucose (**A**) and body weight (**B**) were found in STZ-injected rats, compared with control rats. **C**–**E** Levels of albumin-to-creatinine ratio (ACR), serum creatinine (SCr) and blood urea nitrogen (BUN) in STZ-injected and control rats (n  =  8 for each group). **F** Representative micrographs of histological analysis (PAS staining) of renal tissue from rats with or without STZ treatment. Scale bar: 50 μm. **G** Western blotting analysis of protein (Alix, CD81 and CD63) in urinary small EVs fraction from Ctrl and DN rats. **H** Representative size distribution and particle number of urinary small EVs analyzed by nanoparticle tracking analysis (NTA). The NTA experiment has been performed for three samples in each group. Relative CCL21 mRNA expression in renal cortex (**I**) and urinary small EVs (**J**) from rats with or without STZ injection. **K** The correlation between renal CCL21 mRNA expression and urinary small EVs derived CCL21 mRNA expression in STZ-injected DN rats (r  =  0.6935, p  =  0.0103). *p  <  0.05, **p  <  0.01, ***p  <  0.001, ****p  <  0.0001, compared with Ctrl-rats. *Ctrl* control; *DN* diabetic nephropathy

In order to confirm the upregulation of small EVs derived CCL21 mRNA in DN and its correlation with the level in kidney, we established DN rat model with single injection of STZ. At 20 weeks post injection, the blood glucose of the STZ-injected DN rats increased, and the body weight decreased significantly, compared with the normal group (Fig. 5A, B). The urine ACR, serum creatinine (SCr) and blood urea nitrogen (BUN) in DN rats increased significantly compared with normal group (p  <  0.01; Fig. 5C–E). Histologically, diabetic rats developed significant glomerulosclerosis and mesangial cell proliferation (Fig. 5F). Besides, small EVs from urine of rats was characterized using WB and NTA analysis (Fig. 5G, H).

Further, we found that CCL21 mRNA expression increased remarkably in kidney as well as urinary small EVs in DN rats compared to normal group (Fig. 5I, J; p <  0.0001). Importantly, the expression of CCL21 mRNA in urinary small EVs positively correlated with that level in kidneys of DN rats (Fig. 5K; r = 0.6935; p  =  0.0103). Thus, the data confirmed the up-regulation of CCL21 during DN pathogenesis. Urinary small EVs derived CCL21 may be an indicator for the activation of CCL21 signaling in DN kidney.

## Discussion

In this study, we found that small EVs secretion was increased with enrichment of CCL21 mRNA in urine from DN patients, which suggested active EVs production with specific cargoes in DN. Tubule marker AQP1 and AQP2 were detected in small EVs fraction which suggested that urinary small EVs were at least partly originated from tubule. Tubular injury was a critical component of the early course of DN which contribute to the development of DN [22]. Our previous studies found that exosome secretion was enhanced in tubules when exposed to hypoxia, albuminuria [23, 24]. Thus, active EVs secretion may represent the adaptive response of tubule to external stress under DN condition. Interestingly, a recent study reported that endocytosis was one of the enriched biological processes in incipient DN [9]. In diabetic mice model, increase of albumin filtration and reabsorption at the proximal tubules was also observed with the hyperfiltration status of DN [25]. Hence, endocytosis and EVs release may be activated as the interconnected pathways during the early stage of DN. However, we cannot exclude the other originality of urinary EVs-derived CCL21, such as podocytes, inflammatory cells, which need further investigation.

Further analysis showed that CCL21 mRNA derived from urinary small EVs was extremely upregulated in DN group compared to diabetic patients as well as healthy controls. Moreover, a prominent positively correlation was found between urinary small EVs derived CCL21 and levels of proteinuria. Importantly, CCL21 was efficient early biomarker in discriminating DN patients without eGFR reduction from DM population with superior ability compared to eGFR and proteinuria. Moreover, a pretty high diagnostic accuracy was achieved for distinguishing incipient from overt DN which deserved large cohort study for validation. Diagnostic marker to detect DN at early stage is important for early intervention of the disease and to slow the loss of kidney function. Interestingly, studies of early diagnostic biomarkers of DN have found that tubular injury markers, cystatin-C, angiotensinogen, KIM-1 and NGAL are promising candidates [26]. We here demonstrated that urinary CCL21 as the inflammatory markers might be helpful in identifying early DN. Moreover, previous attempts to identify early DN including urinary proteomics classifier, plasma cytokines and chemokines, metabolites and genetic risk scores [8, 27, 28]. Urinary EVs secretion and their content [29–31], especially miRNAs [32–34], were considered as the potential candidates for novel biomarkers of DN. Our data further supported that urinary EVs might be promising source of new biomarker for early diagnosis of DN. However, one of the limitation of this study is the relatively small sample size of patients enrolled, the diagnostic potential of CCL21 derived from urinary small EVs need to be validated in a larger cohort of patients.

Besides, we found significant correlation between urinary small EVs derived CCL21 and alteration of CCL21 in kidney which also related to severity of tubulointerstitial inflammation in both clinic biopsy samples and kidney of DN animal model. It suggested that profile of transcriptome in urinary EVs may provide a non-invasive approach for detecting histological change of DN. Since DN are commonly diagnosed based solely on clinical assessment, biomarker discovery with pathogenic information are limited [2]. Gudehithlu et al. [35] reported that protein markers in urinary exosomes better represent kidney specific protein alterations than whole urine samples. The data in this study provided the direct evidence that urinary EVs represent a useful approach for detecting changes occurring at tissue level and was helpful in identifying the complex pathophysiological processes responsible for DN.

Culminating evidence suggested that chronic inflammatory state sustained by chronic hyperglycemia, oxidative stress contributed to progressive DN, however, which specific inflammatory proteins contributed to this risk remains unclear [36, 37]. Niewczas et al. [38] examined 194 circulating inflammatory proteins in subjects with type 1 and type 2 diabetes. They identified a kidney risk inflammatory signature (KRIS), consisting of 17 proteins enriched in tumor necrosis factor-receptor superfamily members, that was associated with risk of end-stage renal disease. Recent studies reported that urinary netrin-1, periostin and cyclophilin A showed potential for DN diagnosis at the early stage [39, 40]. Our findings identified that CCL21 mediated inflammation response might be critical to the early development and progression of DN [41]. Interestingly, Vaahtomeri et al. found that the chemokine CCL21 mainly localized in the trans-Golgi network and intracellular vesicles of lymphatic endothelial cells. In vitro, secretion of CCL21-positive vesicles in dendritic cells was triggered by a calcium signal [42]. Thus, CCL21 is likely transported into EVs and participated the process of kidney damage in DN. CCL21 is one of the most important chemokine signaling recognizing CCR7, which controls homing of T cells and dendritic cells to areas of the lymph nodes as well as T cell development and lymphorganogenesis [43, 44]. Indeed, we found significant increased infiltration of both CD4  +  and CD8  +  T lymphocytes in DN group compared to DM, and urine small EV derived CCL21 correlated with the number of infiltrated T cells in kidney. CD4  +  and CD8  +  T cells recruitment and infiltration might contribute to the initiation and progression of immunopathogenesis during the progression of DN respectively [45, 46]. However, the in-depth mechanism of T cell population in the pathogenesis of DN remained to be explored. Hence, we speculated that tubule secreted CCL21 mRNA in EVs and promoted the mRNA transfer between cells, which contributed to more T cell infiltration and activation leading to the inflammatory responses of DN.

## Conclusions

Urinary small EVs derived CCL21 mRNA is upregulated in DN and correlated with the alteration of CCL21 and T cell infiltration in renal tissue, which may serve as early biomarker of renal dysfunction and histological damage. CCL21 mediated T cell infiltration may constitute the key mechanism of chronic inflammation in DN.

## Data Availability

All data generated or analyzed during this study are included in this published article.

## Abbreviations

ACR: Albumin creatinine ratio
AQP: Aquaporin
AUC: Area under the ROC curve
BUN: Blood urea nitrogen
CCL: The (C–C motif) chemokine ligand
CCR: C–C chemokine receptor
CD: Cluster of differentiation
CKD: Chronic kidney disease
CKD-EPI: Chronic Kidney Disease Epidemiology Collaboration
CXCL: The chemokine (C–X–C motif) ligand
DM: Diabetes mellitus
DN: Diabetic nephropathy
eGFR: Estimated glomerular filtration ratio
ESR: Erythrocyte sedimentation rate
EVs: Extracellular vesicles
IFN: Interferon
IL: Interleukin
KIM-1: Kidney injury molecule-1
KRIS: Kidney risk inflammatory signature
NGAL: Neutrophil gelatinase-associated lipocalin
NTA: Nanoparticle tracking analysis
ROC: Receiver operating characteristic
SCr: Serum creatinine
SD rats: Sprague Dawley rats
STZ: Streptozotocin
TNF-a: Tumor necrosis factor-alpha
TNFR: Tumor necrosis factor receptor
UA: Uric acid

## References

[1] Alicic RZ, Rooney MT, Tuttle KR (2017). Diabetic kidney disease: challenges, progress, and possibilities. Clin J Am Soc Nephrol.

[2] Looker HC, Mauer M, Nelson RG (2018). Role of kidney biopsies for biomarker discovery in diabetic kidney disease. Adv Chronic Kidney Dis.

[3] Papadopoulou-Marketou N, Kanaka-Gantenbein C, Marketos N, Chrousos GP, Papassotiriou I (2017). Biomarkers of diabetic nephropathy: a 2017 update. Crit Rev Clin Lab Sci.

[4] Colhoun HM, Marcovecchio ML (2018). Biomarkers of diabetic kidney disease. Diabetologia.

[5] Jha JC, Jandeleit-Dahm KA, Cooper ME (2014). New insights into the use of biomarkers of diabetic nephropathy. Adv Chronic Kidney Dis.

[6] Navarro-Gonzalez JF, Mora-Fernandez C, de Fuentes MM, Garcia-Perez J (2011). Inflammatory molecules and pathways in the pathogenesis of diabetic nephropathy. Nat Rev Nephrol.

[7] Alicic RZ, Johnson EJ, Tuttle KR (2018). Inflammatory mechanisms as new biomarkers and therapeutic targets for diabetic kidney disease. Adv Chronic Kidney Dis.

[8] Coca SG, Nadkarni GN, Huang Y (2017). Plasma biomarkers and kidney function decline in early and established diabetic kidney disease. J Am Soc Nephrol.

[9] Van JA, Scholey JW, Konvalinka A (2017). Insights into diabetic kidney disease using urinary proteomics and bioinformatics. J Am Soc Nephrol.

[10] Lee YH, Kim KP, Park SH (2019). Urinary chemokine C–X–C motif ligand 16 and endostatin as predictors of tubulointerstitial fibrosis in patients with advanced diabetic kidney disease. Nephrol Dial Transplant.

[11] van Niel G, D'Angelo G, Raposo G (2018). Shedding light on the cell biology of extracellular vesicles. Nat Rev Mol Cell Biol.

[12] Miranda KC, Bond DT, McKee M (2010). Nucleic acids within urinary exosomes/microvesicles are potential biomarkers for renal disease. Kidney Int.

[13] Erdbrugger U, Le TH (2016). Extracellular vesicles in renal diseases: more than novel biomarkers?. J Am Soc Nephrol.

[14] Valadi H, Ekstrom K, Bossios A, Sjostrand M, Lee JJ, Lotvall JO (2007). Exosome-mediated transfer of mRNAs and microRNAs is a novel mechanism of genetic exchange between cells. Nat Cell Biol.

[15] Das S, Ansel KM, Bitzer M (2019). The extracellular RNA communication consortium: establishing foundational knowledge and technologies for extracellular RNA research. Cell.

[16] Tkach M, Théry C (2016). Communication by extracellular vesicles: where we are and where we need to go. Cell.

[17] Merchant ML, Rood IM, Deegens JKJ, Klein JB (2017). Isolation and characterization of urinary extracellular vesicles: implications for biomarker discovery. Nat Rev Nephrol.

[18] Feng Y, Lv LL, Wu WJ (2018). Urinary exosomes and exosomal CCL2 mRNA as biomarkers of active histologic injury in IgA nephropathy. Am J Pathol.

[19] Lv LL, Cao YH, Ni HF (2013). MicroRNA-29c in urinary exosome/microvesicle as a biomarker of renal fibrosis. Am J Physiol Renal Physiol.

[20] Thery CWK, Aikawa E, Alcaraz MJ, Anderson JD, Andriantsitohaina R (2018). Minimal information for studies of extracellular vesicles 2018 (MISEV2018): a position statement of the International Society for Extracellular Vesicles and update of the MISEV2014 guidelines. J Extracell Vescicles.

[21] Myllymaki JM, Honkanen TT, Syrjanen JT (2007). Severity of tubulointerstitial inflammation and prognosis in immunoglobulin A nephropathy. Kidney Int.

[22] Tervaert TW, Mooyaart AL, Amann K (2010). Pathologic classification of diabetic nephropathy. J Am Soc Nephrol.

[23] Fiseha T, Tamir Z (2016). Urinary markers of tubular injury in early diabetic nephropathy. Int J Nephrol.

[24] Li ZL, Lv LL, Tang TT (2019). HIF-1alpha inducing exosomal microRNA-23a expression mediates the cross-talk between tubular epithelial cells and macrophages in tubulointerstitial inflammation. Kidney Int.

[25] Lv LL, Feng Y, Wen Y (2018). Exosomal CCL2 from tubular epithelial cells is critical for albumin-induced tubulointerstitial inflammation. J Am Soc Nephrol.

[26] Mori KP, Yokoi H, Kasahara M (2017). Increase of total nephron albumin filtration and reabsorption in diabetic nephropathy. J Am Soc Nephrol.

[27] Fiseha T (2015). Urinary biomarkers for early diabetic nephropathy in type 2 diabetic patients. Biomark Res.

[28] Chen CJ, Liao WL, Chang CT, Lin YN, Tsai FJ (2018). Identification of urinary metabolite biomarkers of type 2 diabetes nephropathy using an untargeted metabolomic approach. J Proteome Res.

[29] Zurbig P, Jerums G, Hovind P (2012). Urinary proteomics for early diagnosis in diabetic nephropathy. Diabetes.

[30] Burger D, Thibodeau J-F, Holterman CE, Burns KD, Touyz RM, Kennedy CRJ (2014). Urinary podocyte microparticles identify prealbuminuric diabetic glomerular injury. J Am Soc Nephrol.

[31] De Shankhajit SK, Hosojima M, Ishikawa T, Kaseda R, Sarkar PYY, Kabasawa H, Iida T, Goto S, Toba K, Higuchi Y, Suzuki Y, Hara M, Kurosawa H, Narita I, Hirayama Y, Ochiya T, Saito A (2017). Exocytosis-mediated urinary full-length megalin excretion is linked with the pathogenesis of diabetic nephropathy. Diabetes.

[32] Kamińska A, Platt M, Kasprzyk J (2016). Urinary extracellular vesicles: potential biomarkers of renal function in diabetic patients. J Diabetes Res.

[33] Xie Y, Jia Y, Cuihua X, Hu F, Xue M, Xue Y (2017). Urinary exosomal microRNA profiling in incipient type 2 diabetic kidney disease. J Diabetes Res.

[34] Jia Y, Guan M, Zheng Z (2016). miRNAs in urine extracellular vesicles as predictors of early-stage diabetic nephropathy. J Diabetes Res.

[35] Ghai V, Wu X, Bheda-Malge A (2018). Genome-wide profiling of urinary extracellular vesicle microRNAs associated with diabetic nephropathy in type 1 diabetes. Kidney Int Rep.

[36] Gudehithlu KP, Garcia-Gomez I, Vernik J (2015). In diabetic kidney disease urinary exosomes better represent kidney specific protein alterations than whole urine. Am J Nephrol.

[37] Navarro-Gonzalez JF, Mora-Fernandez C (2008). The role of inflammatory cytokines in diabetic nephropathy. J Am Soc Nephrol.

[38] Woroniecka KIPA, Mohtat D, Thomas DB, Pullman JM, Susztak K (2011). Transcriptome analysis of human diabetic kidney disease. Diabetes.

[39] Niewczas MA, Pavkov ME, Skupien J (2019). A signature of circulating inflammatory proteins and development of end-stage renal disease in diabetes. Nat Med.

[40] Abdel Ghafar MT, Shalaby KH, Okda HI, Gheit REAE, Soliman NA, Keshk WA (2020). Assessment of two novel renal tubular proteins in type 2 diabetic patients with nephropathy. J Investig Med.

[41] Elkholy RA, Younis RL, Allam AA, Hagag RY, Abdel Ghafar MT (2021). Diagnostic efficacy of serum and urinary netrin-1 in the early detection of diabetic nephropathy. J Investig Med.

[42] Bonventre JV (2012). Can we target tubular damage to prevent renal function decline in diabetes?. Semin Nephrol.

[43] Vaahtomeri K, Brown M, Hauschild R (2017). Locally triggered release of the chemokine CCL21 promotes dendritic cell transmigration across lymphatic endothelia. Cell Rep.

[44] Seeger H, Bonani M, Segerer S (2012). The role of lymphatics in renal inflammation. Nephrol Dial Transplant.

[45] Hauser MA, Legler DF (2016). Common and biased signaling pathways of the chemokine receptor CCR7 elicited by its ligands CCL19 and CCL21 in leukocytes. J Leukoc Biol.

[46] Moon J-Y, Jeong K-H, Lee T-W, Ihm C-G, Lim SJ, Lee S-H (2012). Aberrant recruitment and activation of T cells in diabetic nephropathy. Am J Nephrol.

[47] Mensah-Brown EPK, Obineche EN, Galadari S (2005). Streptozotocin-induced diabetic nephropathy in rats: the role of inflammatory cytokines. Cytokine.

